# HIV-1 Tat protein induces the production of IDO in human monocyte derived-dendritic cells through a direct mechanism: effect on T cells proliferation

**DOI:** 10.1186/1742-4690-10-S1-P113

**Published:** 2013-10-11

**Authors:** Rémi Planes, Elmostafa Bahraoui

**Affiliations:** 1 Université Paul Sabatier, 118 Route de Narbonne, Toulouse, France; 2 INSERM, U1043, CPTP, CHU Purpan, Toulouse, France

## Background

During HIV-1 infection, an increase of indoleamine 2,3 dioxygenase (IDO) expression, and dendritic cells (DC) dysfunction were often associated with AIDS disease progression [1].

## Materials and methods

In this work, we investigated the effect of Tat recombinant protein from HIV-1 Lai and SF-2 strains on the expression of IDO, in Monocyte-derived dendritic cells (MoDCs) generated following 5 days of culture in the presence of GM-CSF and IL-4. IDO expression was analysed by SDS-PAGE and western blotting, intracellular labelling or by measuring kynurenine production by Ehrlich's assay. The capacity of Tat-treated MoDC to stimulate T cell proliferation was analysed by following CFSE dilution in the presence or absence of IDO inhibitor (1-methyl-tryptophane).

## Results

We show that Tat induces IDO protein expression and activity in a dose dependent manner by acting at the cell membrane level. Using different Tat-fragments, we show that the N-Terminal domain, Tat 1-45, but not the central region, Tat 30-72, is sufficient to induce the expression of active IDO. Tat protein is also able to induce several cytokines in MoDCs, including IFN-γ, a strong inducer of IDO. In order to understand whether IDO is induced directly by Tat protein or indirectly following IFN-γ production, complementary experiments were performed and showed that: i) at the kinetic level, Tat induced IDO expression before the production of IFN-γ ii) treatment of MoDCs with Tat-conditioned medium was unable to stimulate IDO expression, iii) coculture of MoDCs in a transwell cell system did not allow IDO expression in MoDCs not previously treated by Tat, iv) direct contact between Tat-treated and untreated MoDCs was not sufficient to induce IDO expression in a Tat-independent manner, and v) treatment of MoDCs in the presence of IFN-γ pathway inhibitors, Jak I and Ly294002, inhibited IFN-γ-induced IDO but had no effect on Tat-induced IDO. At the functional level, our data showed that treatment of MoDCs with Tat led to the inhibition of their capacity to stimulate T cell proliferation. This impairement was totally abolished when the stimulation was performed in the presence of 1MT, an inhibitor of IDO activity, arguing for the implication of the kynurenine pathway.

## Conclusions

By inducing IDO, Tat protein may be considered, as a viral pathogenic factor, in the dysregulation of the DC functions during HIV-1 infection.
